# Human MHC class I molecule, HLA-A2.1, mediates activation of CD8^+^ T cell IFN-γ production and the T cell-dependent protection against reactivation of cerebral *Toxoplasma* infection

**DOI:** 10.3389/fimmu.2022.1005059

**Published:** 2022-10-13

**Authors:** Rajesh Mani, Mohamed H. Abdelaziz, Alexandra Michelon, Yasuhiro Suzuki

**Affiliations:** Department of Microbiology, Immunology and Molecular Genetics, University of Kentucky College of Medicine, Lexington, KY, United States

**Keywords:** HLA-A2.1, humanize mice model, *Toxoplasma gondii*, CD8+ T cell, cerebral infection, chronic infection, toxoplasmic encephalitis

## Abstract

To examine whether the HLA-A2.1, one of the most common MHC class I molecules in humans, activates the protective immunity against reactivation of cerebral infection with *Toxoplasma gondii*, HLA-A2.1-transgenic and wild-type (WT) mice were infected and treated with sulfadiazine to establish chronic infection in their brains. One month after discontinuation of sulfadiazine, which initiates reactivation of the infection, mRNA levels for tachyzoite (the acute stage form)-specific SAG1 and numbers of the foci associated tachyzoites were significantly less in the brains of the HLA-A2.1-transgenic than WT mice. Greater numbers of IFN-γ-producing CD8^+^ T cells were detected in the spleens of infected transgenic than WT mice, and CD8^+^ T cells from the former produced markedly greater amounts of IFN-γ than the T cells from the latter in response to tachyzoite antigens *in vitro*. When their CD8^+^ T cells were systemically transferred to infected immunodeficient NSG mice expressing the HLA-A2.1, the CD8^+^ T cells from HLA-A2.1-transgenic mice inhibited reactivation of the cerebral infection in the recipients more efficiently than did the WT T cells. Furthermore, the inhibition of reactivation of the infection by CD8^+^ T cells from the transgenic mice was associated with increased cerebral expression of IFN-γ and effector molecules against tachyzoites in the recipients when compared to the WT CD8^+^ T cell recipients. Thus, the human HLA-A2.1 is able to effectively activate IFN-γ production of CD8^+^ T cells against *T. gondii* tachyzoites and confer a potent protection against reactivation of cerebral infection with this parasite through the CD8^+^ T cells activation.

## Introduction

*Toxoplasma gondii*, an obligate intracellular protozoan parasite, is an important pathogen that infects not only a variety of mammals including humans (1, 2) but also birds and reptiles (3, 4). During the acute stage of infection, tachyzoites proliferate within nucleated cells (1, 2, 5) and can cause serious diseases including lymphadenopathy and congenital infection to fetuses (1). Whereas IFN-γ-mediated protective immunity limits the tachyzoite growth (6), the tachyzoites convert into bradyzoites and forms tissue cysts especially in the brain and skeletal muscle to establish a chronic infection (5, 7, 8). This infection is abundant not only in developing countries but also in developed countries, and one third of humans are estimated to be chronically infected with this parasite (1). An important issue of this chronic infection in public health is that when the immune system becomes suppressed in the chronically infected individuals by HIV infection, neoplasms, or immunosuppressive treatments for organ transplants, this infection can reactivate and cause life-threatening toxoplasmic encephalitis (TE) (1). TE is the most common opportunistic infectious disease in the brain in AIDS patients (1, 9–11). A rupture of the cysts initiates the reactivation of chronic *T. gondii* infection, which is followed by conversion of released bradyzoites to tachyzoites and proliferation of the tachyzoites. To improve the prevention of TE, it is crucial to define the mechanisms by which the protective immunity prevents the reactivation of chronic infection with *T. gondii*.

Whereas *T. gondii* has three common genotypes, types I, II, and III, the type II is most common in the strains isolated from individuals with TE in Europe and the United States (12–14). Based on this evidence, we developed a murine model of TE, which can mimic the reactivation of *T. gondii* infection in humans, by infecting immunodeficient mice with a type II strain of *T. gondii*. In this model, mice deficient in T cells (e.g. athymic nude or SCID mice) are infected and treated with sulfadiazine, which inhibits tachyzoite growth during the acute stage of infection and leads to a chronic infection in their brains (15). Discontinuation of sulfadiazine induces a reactivation of cerebral *T. gondii* infection in these immunodeficient mice, and this reactivation of infection can be efficiently prevented by an adoptive transfer of immune T cells from infected wild-type (WT) mice (15). With use of this murine model, we previously identified that CD8^+^ T cells play a critical role in the protective immunity to prevent the TE although CD4^+^ T cells also participate (15), and that the protective activity of the CD8^+^ T cells is mediated by their IFN-γ production (16, 17).

CD8^+^ T cells utilizes their T cell receptors to recognize their target antigens presented by the MHC class I molecules expressed on the surface of infected cells, and the different MHC class I molecules can present different antigens to activate the T cells. In mice, the H-2L^d^, one of the MHC class I molecules, can mediate a potent activation of the protective CD8^+^ T cells against *T. gondii* and the prevention of TE (18, 19). In humans, although the HLA-A2, -A11, and -B7 are the three most common MHC class I molecules (20–22), it remains unknown whether any of these most common human MHC class I molecules can mediate an activation of the protective CD8^+^ T cells to prevent TE during *T. gondii* infection. In the present study, we examined whether the human HLA-A2.1 is able to mediate an activation of IFN-γ production by CD8^+^ T cells following *T. gondii* infection and inhibit reactivation of cerebral *T. gondii* infection using transgenic mice expressing the HLA-A2.1. We identified that the HLA-A2.1 is able to induce a robust activation of IFN-γ production by CD8^+^ T cells against *T. gondii* tachyzoites and confer an effective CD8^+^ T cell-mediated protection to prevent cerebral tachyzoite growth induced by reactivation of chronic infection with the parasite.

## Materials and methods

### Mice

C57BL/6-background HLA-A2.1-transgenic (stock #003475) and WT mice (stock #000664) were obtained from the Jackson Laboratories (Bar Harbor, ME). Immunodeficient NSG mice expressing the HLA-A2.1 (stock #014570) were also from the Jackson Laboratory. Swiss-Webster mice were from Taconic (Germantown, NY). Mouse care and experimental procedures were performed under specific pathogen-free conditions in accordance with established institutional guidance and approved protocols from the Institutional Animal Care and Use Committee. Female mice were used for all studies. There were three to seven mice in each experimental group.

### Infection with *T. gondii*

The ME49 strain of *T. gondii* was maintained *in vivo* by infecting Swiss-Webster mice with 10 cysts intraperitoneally, and cysts obtained from their brains during the later stage of infection were used for infection in each study (15, 23). The HLA-A2.1-transgenic and WT mice were infected with 10 cysts intraperitoneally and received sulfadiazine in the drinking water (400 mg/L) beginning at 7 days after infection for 10 days to control tachyzoite proliferation and establish a chronic infection in their brains (15, 24). Discontinuation of sulfadiazine induces reactivation of the chronic cerebral infection due to genetic susceptibility of C57BL/6 mice to the infection (18, 19). Their brains were obtained at 4 to 5 weeks after discontinuation of sulfadiazine treatment, which was the time that some of the WT mice started developing clinical signs of illness (e.g. piloerection, weight loss and/or abnormal [hunched] posture). Three independent studies were performed, with 3-7 mice in each experimental group.

### RNA extraction and real time RT-PCR

RNA was extracted from a half of each brain using RNA STAT-60 (Tel-test, Friendswood, TX) as described previously (25, 26). The RNA concentration was measured using Nanodrop ND-2000 (Thermo Fisher Scientific, Waltham, MA). The purified RNA was first treated with DNase I (Invitrogen, Waltham, MA) to remove genomic DNA contamination. cDNA was synthesized with 1 or 4 μg of the DNase I-treated RNA using reverse transcriptase (Invitrogen) and random hexamers (Invitrogen). The PCR reactions were performed with the cDNA using StepOnePlus real-time PCR system with Taqman reagents (Applied Biosystems, Branchburg, NJ). All primers and probes were from Applied Biosystems. The reagents for mouse IFN-γ (assay ID # Mm01168134), CD8β1 (assay ID #AIFAQ4P), guanylate-binding protein 1 (Gbp1) (assay ID #Mm00657086), indoleamine-2, 3-dioxygenase 1 (IDO1) (assay ID #Mm00492586), inducible nitric oxide synthase (NOS2) (assay ID #Mm00440502), immunity-related GTPase family member m3 (Irgm3) (assay ID #Mm00497611), perforin (Prf1) (assay ID #Mm00812512), and β-actin (a house-keeping gene) (assay ID #4352933E) were their ready-made products. The sequences of primers and probes for *T. gondii* tachyzoite-specific SAG1 and bradyzoite (cyst)-specific BAG1 are as follows: 5’- CACAGAGTTGTATGGTCACA GTGA -3’ (forward), 5’- GCACCGTAGGAGCACCTT-3’ (reverse), and 5’- TCGGTCGTCAATAATG -3’ (probe) for SAG1 (27); and 5’-TCACGTGGAGACCCAGAGT-3’ (forward), 5’-CTGGCAAGTCAGCCAAAATAATCAT-3’ (reverse), and 5’-TTTGCTGTCGAACTCC-3’ (probe) for BAG1 (26). Amounts of mRNA for these molecules were normalized to amounts of mRNA for β-actin.

### Immunohistochemical analyses to detect the foci associated with *T. gondii* tachyzoites

Sagittal sections (4μm thickness) of the brains were deparaffinized and rehydrated using xylene (Sigma-Aldrich, St. Louis, MO), ethanol (Decon Labs, King of Prussia, PA), and then water. Heat-induced epitope retrieval was performed in citrate buffer (pH 6) within a microwave for 5 minutes. Thereafter, the slides were treated with 3% hydrogen peroxide solution for 15 minutes to block endogenous peroxidase activity, followed by blocking with 5% bovine serum albumin (Sigma-Aldrich) in Tris-buffered saline with 0.5% Tween 20 (TBST) for 2 hours. The slides were then incubated with rabbit anti-*T. gondii* antibodies for 1 hr at a room temperature. The slides were washed in TBST and incubated with horseradish peroxidase-conjugated goat anti-rabbit IgG antibody (Invitrogen) for one hour at room temperature. Color was developed using diaminobenzidine (Vector Laboratories, Burlingame, CA). These slides were then stained with hematoxylin to visualize the nuclei of host cells. Numbers of foci associated with tachyzoites and numbers of cysts in the entire field of each sagittal section were counted microscopically using Nikon Eclipse 90i microscope, and images were obtained using a digital camera and NIS elements BR 3.2 software (Nikon). Seven sections (at least 40 μm distance between sections) were analyzed for each mouse in one set of the experiment that had 4 mice in each experimental group. Four sections for each mouse were analyzed in another set of the experiment with 8 or 11 mice in each experimental group. Two individuals performed the analyses on 3 or 4 sections, 7 sections in total, in each mouse independently in the first set of the experiment. In the second set of the experiment, each of them analyzed 2 sections, 4 in total, on each mouse in the same manner. The mean value of those counts from the two individuals on each mouse was used for the analysis.

### Purification of CD8^+^ T cells and measuring the frequencies of IFN-γ-producing CD8^+^ T cells and the TCR Vβ chain usages of those T cells

Spleen cells were obtained from two mice from each of infected HLA-A2.1-transgenic and WT mouse groups at 4-5 weeks after infection and suspended in Hank’s balanced salt solution (HyClone [cytiva], Mariborough, MA) with 2% fetal bovine serum (Sigma-Aldrich). The spleen cells were pooled within the same experimental group, and CD8^+^ T cells were purified from these spleen cells using magnetic beads conjugated anti-mouse CD8α (clone 53-6.7) monoclonal antibodies (mAbs) (Miltenyi Biotech, Auburn, CA) and MACS column (Miltenyi) (17). The purified CD8^+^ T cells were then stimulated with 5 ng/ml phorbol myristate acetate (Sigma) and 500 ng/ml ionomycin (Sigma-Aldrich) in the presence of Golgi Plug (BD Biosciences, Mountain View, CA) as previously described (24, 28). After the stimulation, the cells were incubated with APC-conjugated anti-CD8α mAb (clone 53-6.7) in combination with FITC-conjugated mAbs to 15 different T cell receptor (TCR) Vβ chains, followed by fixation and permeabilization with Cytofix/Cytoperm Plus kit (BD Biosciences) and stained with PE-conjugated anti**-**IFN-γ (XMG1.2) or isotype control (R3-34) mAbs (24). All mAbs were purchased from BD Biosciences. Cells were analyzed on BDSymphony A3 using DIVA software (BD Biosciences).

### CD8^+^ T cell cultures and their IFN-γ production in response to *T. gondii* tachyzoite antigens

The CD8^+^ T cells were purified from the spleens of four mice in each of HLA-A2.1-transgenic and WT mouse groups at three weeks after infection. The purified CD8^+^ T cells were placed in 96-well plates at a density of 3×10^5^ cells per well that contains spleen cells (1.5 x 10^5^ cells) from infected, sulfadiazine-treated immunodeficient NSG mice expressing HLA-A2.1 as antigen-presenting cells. The NSG mice lack both T and B cells and NK cells. The tachyzoite lysate antigens (TLA) (10 μg/ml) were added to a part of these wells. There were 4 wells in each experimental group for both with and without the TLA. After 72 hrs of incubation, the culture plates were centrifuged at 800 rpm for 10 min at 4 °C and the culture supernatants were collected. The concentrations of IFN-γ in the culture supernatants were measured by ELISA using a kit from BD Biosciences (29).

### Adoptive transfer of CD8^+^ T cells from infected HLA-A2.1-transgenic and WT mice to infected NSG mice expressing HLA-A2.1.

CD8^+^ T cells were purified from the spleens of infected HLA-A2.1-transgenic or WT mice at 4 weeks after discontinuation of their sulfadiazine treatment. There were four mice in each of these donor groups. As recipients of these CD8^+^ T cells, immunodeficient NSG mice expressing HLA-A2.1 were infected intraperitoneally with 10 cysts and received sulfadiazine in drinking water beginning at 5 days after infection. At three to four weeks after infection, these mice were injected intravenously from a tail vein with CD8^+^ immune T cells (2 x 10^6^ cells) purified from the spleens of infected HLA-A2.1-transgenic or WT mice. Five days after the CD8^+^ T cell transfer, sulfadiazine treatment on the recipients was discontinued to initiate reactivation of cerebral infection with *T. gondii*, and 4 days later, their brains were collected for analyses.

### Statistical analysis

Levels of significance in differences between experimental groups in mRNA levels for SAG1 and BAG1 of *T. gondii*, IFN-γ, and effector molecules in IFN-γ-mediated protective immunity, numbers of foci associated with tachyzoites, and IFN-γ levels in the culture supernatants were determined by Student’s *t* or Mann-Whitney *U* test. These analyses were performed using GraphPad Prism software 9.0. Differences that had *p* values < 0.05 were considered significant.

## Results

### Expression of human HLA-A2.1 molecule confers a marked protection against reactivation of *T. gondii* infection in the brain in genetically susceptible C57BL/6 ;mice

**Figure 1:**
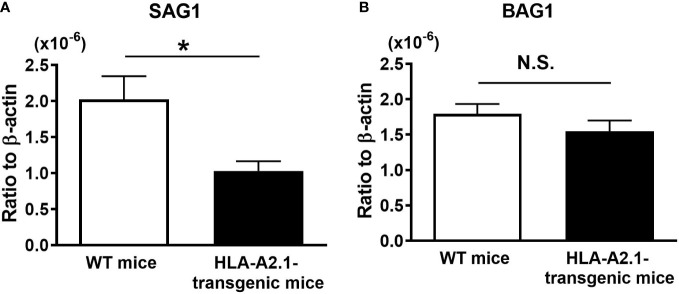
Transgenic mice expressing human HLA-A2.1-have lower tachyzoite burden in their brains than do WT control mice during reactivation of cerebral infection with *T. gondii*. The HLA-A2.1-transgenic and WT control mice were infected with 10 cysts of the ME49 strain and treated with sulfadiazine beginning at 7 days after infection for 10 days to control tachyzoite proliferation during the acute stage of infection and establish chronic infection in their brains. Four to five weeks after the discontinuation of sulfadiazine, which induces reactivation of the cerebral infection, the ratios of mRNA levels for **(A)** tachyzoite-specific SAG1 and **(B)** bradyzoite (cyst)-specific BAG1 against mRNA levels for β-actin were measured by real-time RT-PCR on the brains. *P<0.05. N.S. Not significant. The data were combined from three independent experiments (n=11 for the HLA-transgenic mice, and n=14 for the WT control mice).

To examine whether the HLA-A2.1 confers a protection against reactivation of cerebral *T. gondii* infection, we employed a mouse strain that expresses this human MHC class I molecule in the C57BL/6-background. Since C57BL/6 is one of the strains of mice genetically susceptible to cerebral *T. gondii* infection (18, 19), the expression of the HLA-A2.1 in these mice is suitable for examining whether this human MHC class I molecule confers a protection against the infection. HLA-A2.1-transgenic and WT C57BL/6 mice were infected and treated with sulfadiazine to inhibit tachyzoite growth during the early stage of infection and establish chronic cerebral infection. Discontinuation of sulfadiazine initiates reactivation of the infection in their brains. Four to five weeks after the discontinuation of sulfadiazine treatment, which is the time that some of the WT mice started developing clinical signs of illness such as piloerection and abnormal posture (hunched), we measured tachyzoite burdens in the brains of these two strains of mice. Tachyzoite-specific SAG1 mRNA levels in the brains of the HLA-A2.1-transgenic mice were approximately a half of those in the brains of the WT mice (*P*<0.05) (**Figure 1A**). In contrast, amounts of mRNA for bradyzoite (cyst)-specific BAG1 did not differ between these two strains of mice (**Figure 1B**). These results indicate that the presence of the human MHC class I molecule, HLA-A2.1, confers a significant protection against cerebral tachyzoite proliferation caused by reactivation of chronic infection with *T. gondii*.

**Figure 2:**
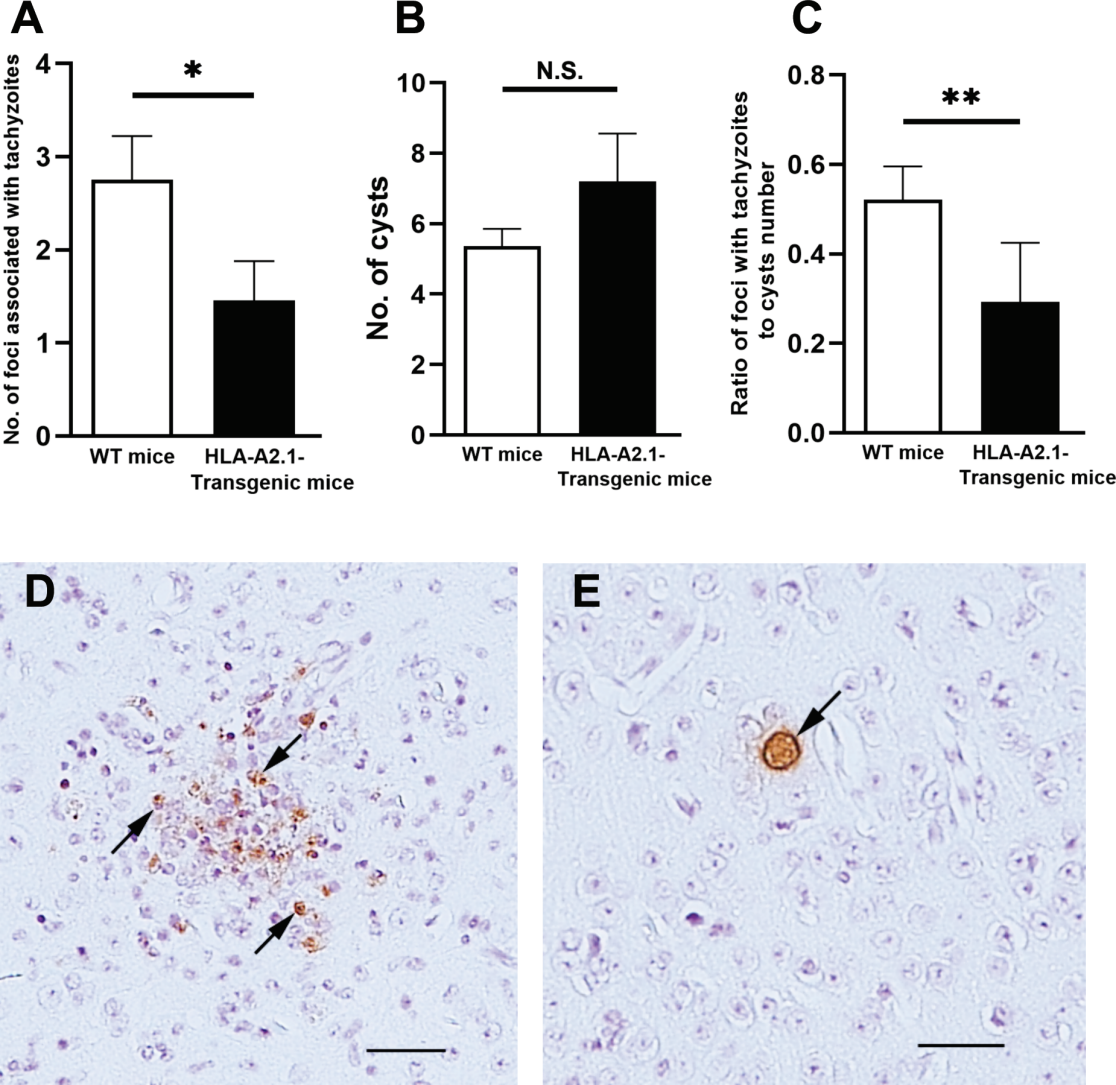
The HLA-A2.1-transgenic mice have fewer areas of foci associated with tachyzoites than do WT control mice in their brains during reactivation of cerebral infection with *T. gondii*. The HLA-A2.1-transgenic and WT control mice were infected with 10 cysts of the ME49 strain and treated with sulfadiazine beginning at 7 days after infection for 10 days to control tachyzoite proliferation during the acute stage of infection and establish chronic infection in their brains. Four to five weeks after discontinuation of sulfadiazine, which induces reactivation of the cerebral infection, immunohistochemical analyses staining for the parasite were performed on the sagittal sections (4 μm thickness) of their brains, and numbers of the foci associated with tachyzoites and numbers of the cysts were microscopically counted in the entire filed of each sagittal section. A total of 4 or 5 sections (at least 40 μm distance between sections) were analyzed for each mouse, and the mean value from the counts from those sections were calculated for each of the number of **(A)** tachyzoite-associated foci and **(B)** the cysts, and **(C)** ratios of the tachyzoite-associated foci number to the cyst number for each mouse. **(D)** A representative image of the foci associated with tachyzoites (some representative tachyzoites are allowed) and an accumulation of inflammatory cells. **(E)** A representative image of the cysts (the cyst wall is allowed). The bars in the panels D and E indicate the distance of 50 μm. *P<0.05, **P<0.01. N.S. Not significant. The data were combined from two independent experiments (n= 12 for the HLA-transgenic mice, and n=15 for the WT control mice).

We also performed immunohistochemical analyses to compare the numbers of foci associated with tachyzoites in the brains of the HLA-A2.1-transgenic and WT mice during the reactivation of *T. gondii* infection. Consistent with the amounts of mRNA for tachyzoite-specific SAG1, numbers of foci associated with tachyzoites in the brains of the HLA-A2.1-transgenic mice were approximately a half of those of the WT mice (*P*<0.05) (**Figure 2A**). A representative image of those foci associated with many tachyzoites is shown in **Figure 2D** (some tachyzoites are arrowed). An accumulation of large numbers of inflammatory cells was associated with those areas of tachyzoite growth (**Figure 2D**). In contrast, cyst numbers detected in their brains did not differ between these two strains (**Figure 2B**), which is consistent with the presence of similar levels of bradyzoite-specific BAG1 mRNA in the brains of these groups of mice as shown in **Figure 1B**. A representative image of a cyst is shown in **Figure 2E**, with an arrow indicating the presence of cyst wall surrounding numbers of bradyzoites. Inflammatory cells were not detected around the cysts (**Figure 2E**). Notably, the ratios of the number of foci associated with tachyzoites to the number of cysts in the infected HLA-A2.1-transgenic mice were less than a half of those of the WT mice (*P*<0.01) (**Figure 2C**). These results further confirm that the presence of the HLA-A2.1 confers an effective protection against cerebral tachyzoite growth associated with reactivation of chronic *T. gondii* infection.

### Efficiency of expression of IFN-γ and effector molecules in the IFN-γ-mediated protective immunity in response to tachyzoite growth in the brain is greater in HLA-A2.1-transgenic than WT mice during reactivation of *T. gondii* infection

**Figure 3:**
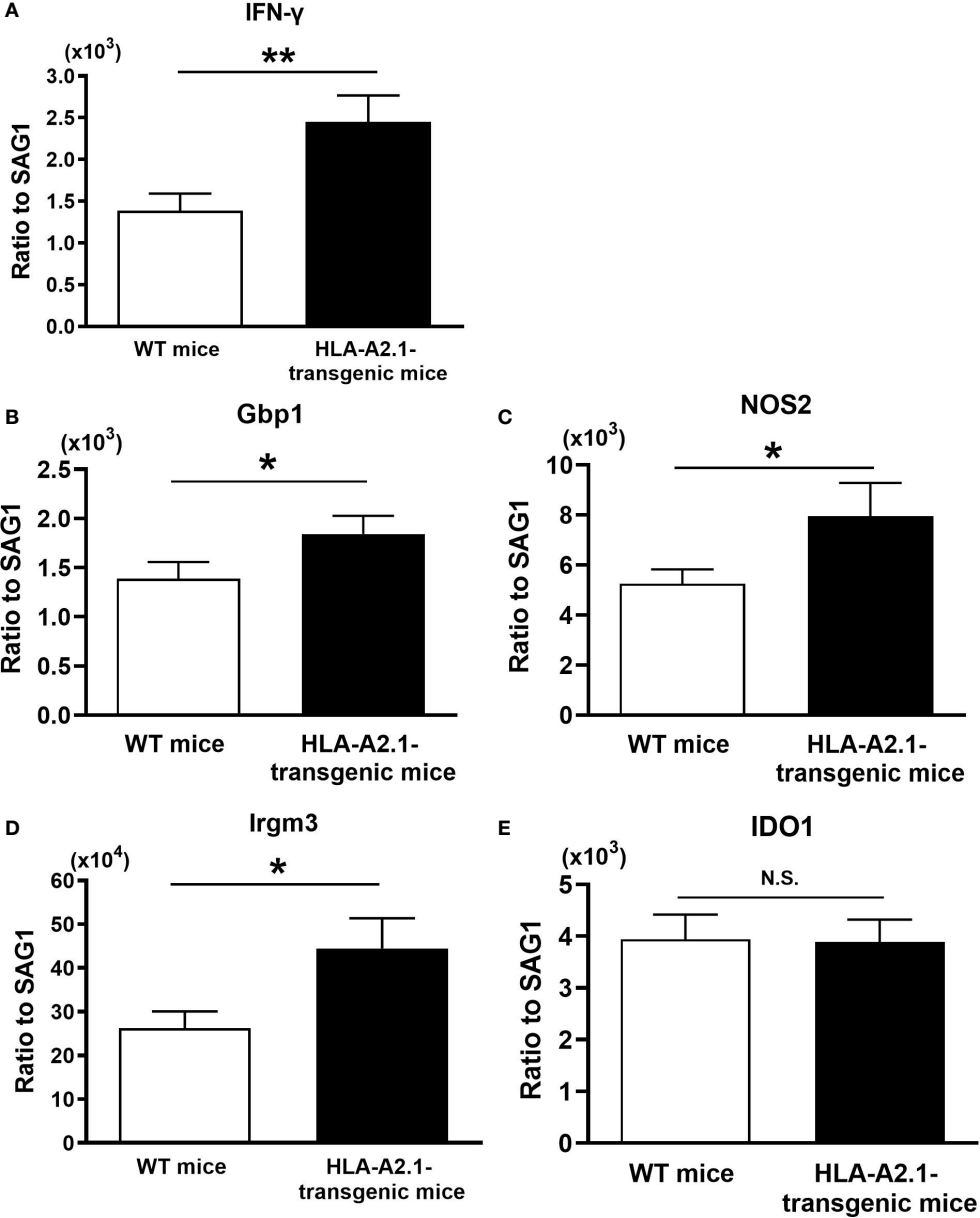
The efficiency of expressions of IFN-γ and effector molecules for the IFN-γ-mediated protective immunity against tachyzoites are higher in the brains of the HLA-A2.1-transgenic than those of WT control mice during reactivation of cerebral infection with *T. gondii*. The HLA-A2.1-transgenic and WT control mice were infected with 10 cysts of the ME49 strain and treated with sulfadiazine beginning at 7 days after infection for 10 days to control tachyzoite proliferation during the acute stage of infection and establish chronic infection in their brains. Four to five weeks after the discontinuation of sulfadiazine, which induces reactivation of the cerebral infection, the efficiency of the expression of mRNA for **(A)** IFN-γ, **(B–E)** the effector molecules (Gbp1, NOS2,Irgm3, and IDO1) in the IFN-γ-mediated protective immunity in response to tachyzoites (the ratios of mRNA levels for IFN-γ and the effector molecules against mRNA levels for tachyzoite-specific SAG1) were measured by real-time RT-PCR on the brains. *P<0.05, **P<0.01. N.S. Not significant. The data were combined from three independent experiments (n= 10 or 11 for the HLA-transgenic mice, and n=14 for the WT control mice).

IFN-γ is required for the host defense to prevent cerebral tachyzoite growth (30, 31), and CD8^+^ T cells play a key role in the protective immunity to prevent TE (15). Since the HLA-A2.1 is a human MHC class I molecule that presents target antigens to CD8^+^ T cells, it is likely that the increased resistance of HLA-A2.1-transgenic mice against reactivation of cerebral *T. gondii* infection is mediated by an activation of the protective CD8^+^ T cells through the antigen presentation by this human MHC class I molecule. When CD8^+^ T cells migrate into the brain, they need to recognize their target *T. gondii* antigens for activating their IFN-γ production. Therefore, their IFN-γ production will depend on not only their capability to produce this cytokine but also the frequencies of target *T. gondii* antigens present in the brain. If larger numbers of tachyzoites are present, the recruited T cells will have greater chances to be activated by recognizing their target tachyzoite antigens and produce IFN-γ. Therefore, for comparing the efficiency of the T cells to produce IFN-γ in the brains of infected HLA-A2.1-transgenic and WT mice during reactivation of the infection, we measured the ratios of IFN-γ mRNA levels to tachyzoite-specific SAG1 mRNA levels in their brains at 4-5 weeks after discontinuation of sulfadiazine treatment. The IFN-γ mRNA/SAG1 mRNA ratios were approximately twice greater in the HLA-A2.1-transgenic than WT mice (*P*<0.01) (**Figure 3A**).

Gbp1, NOS2, Irgm3, and IDO1 have been shown to function as effector molecules in the IFN-γ-mediated protective immunity to control *T. gondii* tachyzoites (32, 33). Therefore, we also compared the efficiencies of cerebral expression of these effector molecules against tachyzoites in the brains of the HLA-A2.1-transgenic and WT mice during reactivation of the infection. The ratios of the amounts of mRNA for each of Gbp1, NOS2, and Irgm3 to SAG1 mRNA levels were significantly greater in the brains of the HLA-A2.1-transgenic than WT mice (*P*< 0.05) (**Figures 3B–D**), although the ratio of IDO1 mRNA levels to SAG1 mRNA levels did not differ between these two strains of mice (**Figure 3E**). These results strongly suggest that the enhanced protection of infected HLA-A2.1-transgenic mice against reactivation of cerebral *T. gondii* infection when compared to the WT mice is mediated by the increased efficiency of IFN-γ production by CD8^+^ T cells against tachyzoites and an activation of the IFN-γ-mediated protective immunity against the parasite in their brains.

In contrast to the protective immunity against tachyzoites, the immune system utilizes perforin-mediated cytotoxic activities of CD8^+^ T cells to remove *T. gondii* cysts (25, 34). Consistent with the presence of equivalent levels of bradyzoite-specific BAG1 mRNA and cyst numbers in the brains of infected HLA-A2.1-transgenic and WT control mice shown in **Figures 1B** and **2B**, the ratios of perforin (Prf1) mRNA levels to BAG1 mRNA levels did not differ between the brains of these two strains of mice (0.687 ± 0.074 in the HLA-A2.1-transgenic mice [n=12] vs.0.851 ± 0.247 in the WT mice [n=14], *P*=0.274).

### HLA-A2.1-transgenic mice infected with *T. gondii* have greater numbers of IFN-γ-producing CD8^+^ T cells in their spleens than do infected WT mice

**Figure 4:**
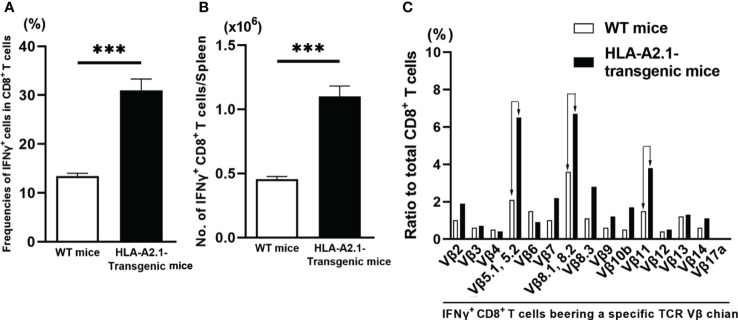
*T. gondii*-infected HLA-A2.1-transgenic mice have larger numbers of IFN-γ-producing CD8^+^ T cells than do infected WT control mice in association with increased usages of TCR Vβ5, Vβ8.1, 8.2, and Vβ11 in their IFN-γ-producing CD8^+^ T cells. CD8^+^ T cells were purified from the spleens of HLA-A2.1-transgenic and WT control mice at 4-5 weeks after infection with *T. gondii*. **(A)** The frequencies and **(B)** numbers of IFN-γ^+^ cells in the CD8^+^ T cells were measured by flow cytometry. **(C)** The usages of TCR Vβ chains among the IFN-γ^+^CD8^+^ T cells were also measured by flow cytometry. ***P<0.001.

To obtain a direct evidence that CD8^+^ T cells of HLA-A2.1-transgenic mice have greater IFN-γ responses to *T. gondii* infection than those T cells of WT mice do, we examined the numbers of IFN-γ-producing CD8^+^ T cells in the spleens of HLA-A2.1-transgenic and WT mice using flow cytometry at 4-5 weeks after infection. The frequencies of IFN-γ producing cells among a total CD8^+^ T cell population were 2.4 times higher in the infected transgenic than WT mice (*P*< 0.001) (**Figure 4A**). A total number of IFN-γ^+^CD8^+^ T cells in their spleens were also more than two-fold higher in the former than the latter (*P*<0.001) (**Figure 4B**). Therefore, the presence of the human HLA-A2.1 induces a markedly enhanced activation of IFN-γ production by CD8^+^ T cells during *T. gondii* infection.

The TCRs expressed on the surface of CD8^+^ T cells recognize their target antigens presented by the MHC class I molecules, and the variable regions of TCR α and β chains determine the specificity of antigen recognition of the TCRs. Therefore, if CD8^+^ T cells potently activate their IFN-γ production by recognizing *T. gondii* antigens presented by the HLA-A2.1, IFN-γ-expressing CD8^+^ T cells of infected HLA-A2.1 transgenic mice would most likely have unique TCR Vβ usages when compared to those of IFN-γ-producing CD8^+^ T cells of infected WT mice. Therefore, we compared the usages of 15 different TCR Vβ chains in IFN-γ^+^CD8^+^ T cells in the spleens of infected HLA-A2.1-transgenic and WT mice using flow cytometry. Markedly increased frequencies of three TCR Vβ chains, Vβ5.1, 5.2, Vβ8.1, 8.2, and Vβ11, were detected in the IFN-γ^+^CD8^+^ T cells of the infected HLA-A2.1-transgenic mice when compared to those of WT mice (**Figure 4C**). The frequencies of the usages of TCR Vβ7 and Vβ8.3, also tended to be greater in the former than the latter (**Figure 4C**). Therefore, it is likely that the TCRs containing Vβ5.1, 5.2, Vβ8.1, 8.2, and Vβ11 expressed on the surface of CD8^+^ T cells contribute to their recognition of *T. gondii* antigens presented by the HLA-A2.1 molecule and activating their IFN-γ production. These results further support that CD8^+^ T cells potentiate their IFN-γ production by recognizing *T. gondii* antigens presented by human HLA-A2.1 molecule.

### CD8^+^ T cells produce large amounts of IFN-γ in response to *T. gondii* tachyzoites presented by the HLA-A2.1

**Figure 5:**
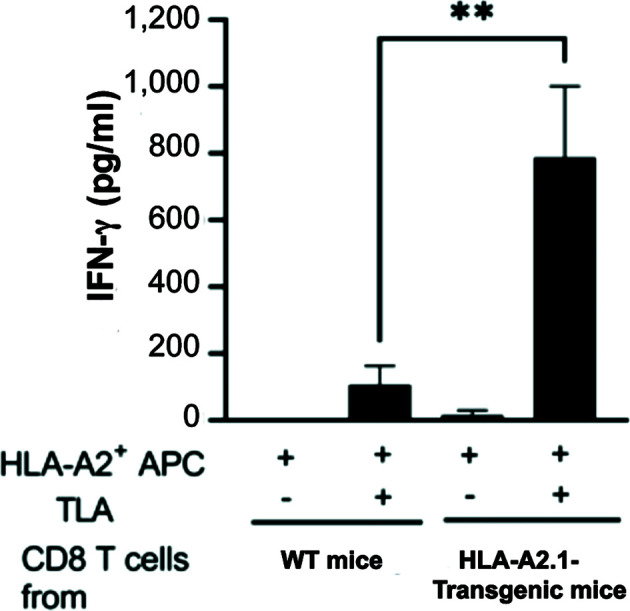
CD8^+^ T cells from *T. gondii*-infected HLA-A2.1-transgenic mice produce larger amounts of IFN-γ than do the T cells from infected WT control mice in response to tachyzoite antigens in the presence of antigen-presenting cells expressing HLA-A2.1. CD8^+^ T cells were purified from the spleens of four mice in each of the HLA-A2.1 and WT mouse groups at 3 weeks after infection, and those T cells (3 x 10*^5^* cells) were stimulated with total tachyzoite lysate antigens (TLA) (10 μg/ml) in the presence of the HLA-A2.1-expressing antigen-presenting cells (1.5 x 10^5^ cells) for 72 hrs. The amounts of IFN-γ in the culture supernatants were measured by ELISA. **P<0.01.

To elucidate the increased capability of CD8^+^ T cells of HLA-A2.1-transgenic mice to produce IFN-γ specifically against tachyzoites that causes reactivation of *T. gondii* infection, CD8^+^ T cells purified from the spleens in the infected HLA-A2.1-transgenic and WT mice were stimulated with total tachyzoite lysate antigens (TLA) *in vitro* in the presence of antigen-presenting cells expressing the HLA-A2.1 (spleen cells of the HLA-A2.1-expressing immunodeficient NSG mice (deficient in T and B cells and NK cells) for 72 hrs. IFN-γ levels in the culture supernatants of the HLA-A2.1-transgenic CD8^+^ T cells in response to TLA were more than 7 times higher than those of the culture of the T cells from the WT mice stimulated with TLA (*P*< 0.01) (**Figure 5**). In contrast, IFN-γ levels in the supernatants of the cultures in the absence of TLA remained very low in the T cells from the both groups of mice (**Figure 5**).

The MHC haplotype of the NSG mice used for antigen-presenting cells is the H2K^g7^, whereas the MHC haplotype of C57BL/6 mice is the H2^b^. The difference between the MHC haplotypes between NSG and C57BL/6 mice is most likely the reason why the CD8^+^ T cells from infected WT C57BL/6 mice produced only small amounts of IFN-γ in response to tachyzoite antigens presented by antigen-presenting cells from the HLA-A2.1-expressing NSG mice. Thus, the majority of IFN-γ produced by the CD8^+^ T cells from the HLA-A2.1-transgenic mice was through recognition of tachyzoite antigens presented by the HLA-A2.1.

### Adoptive transfer of CD8^+^ immune T cells confers a powerful protection against reactivation of cerebral *T. gondii* infection through the HLA-A2.1

**Figure 6:**
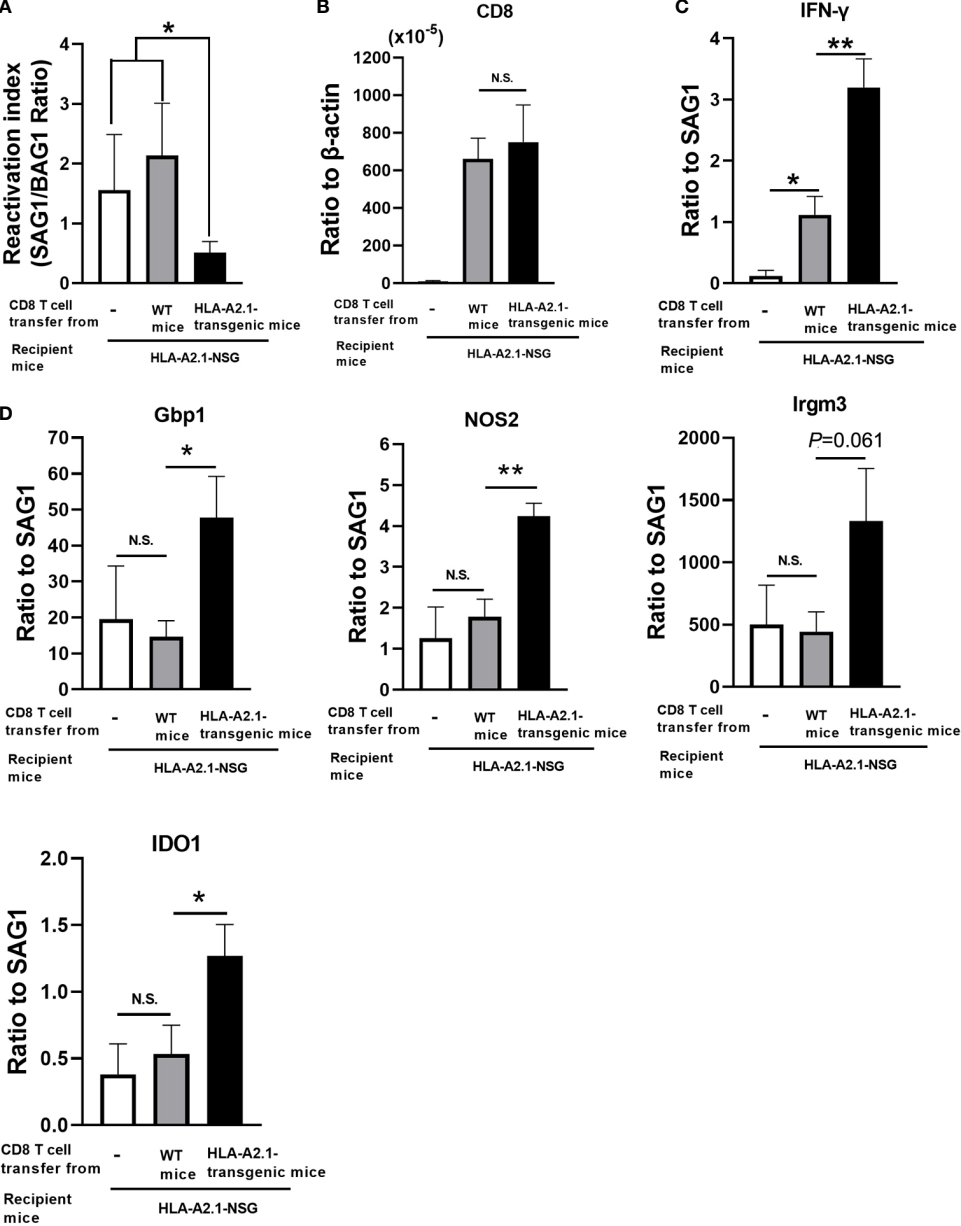
Adoptive transfer of CD8^+^ T cells from *T. gondii*-infected HLA-A2.1-transgenic mice confers a greater protection against reactivation of cerebral infection with the parasite in infected, immunodeficient NSG mice expressing the HLA-A2.1 than do a transfer of the T cells from infected WT control mice. CD8^+^ T cells were purified from the spleens of infected HLA-A2.1-transgenic and WT mice at 4 weeks after discontinuation of sulfadiazine. These CD8^+^ T cells (2 x 10^6^ cells) were injected intravenously from a tail vein into infected, sulfadiazine-treated immunodeficient NSG mice expressing the HLA-A2.1 at 4 weeks after infection. An additional group of the HLA-A2.1-expressing NSG mice did not receive any T cells as a control. Five days after the T cell transfer, sulfadiazine treatment on the recipients was discontinued to initiate reactivation of the infection. Four days after the initiation of reactivation of the infection, **(A)** the reactivation index (ratios of the SAG1 mRNA levels to the BAG1 mRNA level), **(B)** the ratios of mRNA levels for CD8β to mRNA levels for β-acting, and the ratios of mRNA levels for **(C)** IFN-γ and **(D)** IFN-γ-mediated effector molecules (Gbp1, NOS2, Irgm3, and IDO1) to mRNA levels for tachyzoite-specific SAG1 were measured by real-time RT-PCR on the brains. *P<0.05, **P<0.01. N.S. Not significant.

To demonstrate that CD8^+^ T cells activated by recognition of *T. gondii* antigens presented by the HLA-A2.1 confer a protection against reactivation of infection with this parasite, CD8^+^ immune T cells (2 x 10^6^ cells) purified from the spleens of infected HLA-A2.1-transgenic and WT mice were systemically transferred to infected, sulfadiazine-treated immunodeficient NSG mice expressing the HLA-A2.1. In this way, the differences in the protective activity of the transferred CD8^+^ T cells from the two donor groups against reactivation of *T. gondii* infection in the recipients are solely due to the presence or absence of the capability of the transferred CD8^+^ T cells to respond to *T. gondii* antigens presented by the HLA-A2.1 molecule. As an additional control, another group of the infected HLA-A2.1-expressing NSG mice did not receive any T cells. Five days after the T cell transfer, sulfadiazine on the recipient animals was discontinued to initiate reactivation of the infection. Since the reactivation of cerebral *T. gondii* infection is initiated by cyst ruptures that induce proliferation of tachyzoites, a ratio of SAG1 mRNA levels versus BAG1 mRNA levels is a useful indicator of the efficiency of the protective immunity to prevent reactivation of the infection. Five days after the initiation of reactivation of the infection, the reactivation index (the ratio of SAG1 mRNA levels versus BAG1 mRNA levels) in the brains of the recipients of CD8^+^ immune T cells from the HLA-A2.1-transgenic mice were more than three times lower than those of the two control groups with a transfer of no T cells or WT CD8^+^ T cells (*P*<0.05 to the two control groups combined) (**Figure 6A**).

We also compared the efficiency of IFN-γ production by the transferred CD8^+^ T cells in response to tachyzoites (the ratios of IFN-γ mRNA levels vs. SAG1 mRNA levels) in the brains of the CD8^+^ T cell recipients. The ratios of mRNA levels for IFN-γ to those for SAG1 were three times greater in the recipients of CD8^+^ T cells from the HLA-A2.1-transgenic mice than the recipients of the WT T cells (*P*<0.01) (**Figure 6C**). This difference was not due to a difference in the migration of the transferred T cells into the brains of the recipients, since amounts of CD8 mRNA did not differ between the recipients of the transgenic and WT T cells (**Figure 6B**). Since the only difference between the CD8^+^ immune T cells from the HLA-A2.1-transgenic and WT mice is the presence or absence of the capability to respond to *T. gondii* antigens presented by the HLA-A2.1 molecule as shown in **Figure 5**, these results indicate that CD8^+^ immune T cells activated through presentation of *T. gondii* antigens by the HLA-A2.1 are able to produce large amounts of IFN-γ in the brains of the recipients mice by recognizing tachyzoite antigens presented by this human MHC class I molecule.

We also examined the expression of the effector molecules, Gbp1, NOS2, Irgm3, and IDO1 of the IFN-γ-mediated protective immunity against tachyzoites in the brains of the HLA-A2.1-expressing NSG mice that had received CD8^+^ immune T cells from the HLA-A2.1-transgenic and WT mice. The ratios of the mRNA levels for Gbp1, NOS2, and IDO1 to tachyzoite-specific SAG1 mRNA levels were significantly greater in the brains of the recipients of the HLA-A2.1-transgenic CD8^+^ T cells than those of the WT T cells (*P*<0.01 for NOS2, and *P*<0.05 for Gbp1 and IDO1) (**Figure 6D**). The ratio of the Irgm3 mRNA levels to the SAG1 mRNA levels also tended to be greater in the former than the latter, but the difference did not reach statistical significance (*P*=0.061) (**Figure 6D**). These results together indicate that CD8^+^ T cells activated against *T. gondii* by antigen presentation by the HLA-A2.1 are able to potently activate IFN-γ-mediated protective immunity against the parasite through antigen presentation by the HLA-A2.1 in the brains of infected mice and confer a powerful protection against reactivation of cerebral infection with this parasite.

## Discussion

The present study using transgenic mice expressing human HLA-A2.1 molecule uncovered that the HLA-A2.1, one of the most common MHC class I molecules in humans, can mediate a potent protection against reactivation of cerebral *T. gondii* infection. C57BL/6 mice are genetically susceptible to the development of toxoplasmic encephalitis due to cerebral proliferation of tachyzoites during later stage of infection with *T. gondii* (18, 19). We discovered that the expression of the human HLA-A2.1 in the susceptible C57BL/6-background markedly reduces tachyzoite burden and numbers of foci associated with tachyzoites in the brain during reactivation of *T. gondii* infection. Thus, the human HLA-A2.1 molecule is able to effectively mediate a protection against cerebral tachyzoite growth and reactivation of *T. gondii* infection. To our knowledge, the capability of the HLA-A2.1 to confer a protection against cerebral toxoplasmosis has not been reported before.

IFN-γ production by CD8^+^ T cells plays a critical role in the protective immunity to prevent cerebral tachyzoite growth (17, 24, 31). The MHC class I molecules present target antigens to CD8^+^ T cells for their activation. The present study revealed that CD8^+^ immune T cells from infected HLA-A2.1-transgenic mice produced more than 7 times greater amounts of IFN-γ than CD8^+^ T cells from the WT control mice in response to *T. gondii* tachyzoite antigens in the presence of antigen-presenting cells expressing the HLA-A2.1 *in vitro*, whereas IFN-γ production of these T cells remained minimal in the absence of the tachyzoite antigens. Thus, the human HLA-A2.1 is able to present tachyzoite antigens of *T. gondii* to potently activate IFN-γ production of CD8^+^ T cells. This *in vitro* finding is consistent with the *in vivo* evidence on markedly greater ratios of IFN-γ mRNA levels to tachyzoite-specific SAG1 mRNA levels in the brains of infected HLA-A2.1-transgenic than WT mice.

In contrast to the efficient protective effects of the immune responses mediated by antigen presentation by the HLA-A2.1 against cerebral tachyzoite growth, cyst burden in the brains of infected HLA-A2.1-transgenic mice did not differ from that of infected WT control mice. The immune system employs a perforin-mediated cytotoxic activity of CD8^+^ T cells to eliminate the cyst form of *T. gondii* (25, 34), whereas the protective immune responses against tachyzoites are mediated by IFN-γ as mentioned earlier. The present study revealed that the ratios of perforin (Prf1) mRNA levels to bradyzoite-specific BAG1 mRNA levels did not differ between the brains of infected HLA-A2.1-transgenic and WT control mice, which is a clear contrast to the presence of greater ratios of IFN-γ mRNA levels to tachyzoite-specific SAG1 mRNA levels in the brains of the former than the latter. Therefore, the HLA-A2.1 molecule may be able to presents tachyzoite antigens to activate IFN-γ production of CD8^+^ T cells more effectively than it presents cyst antigens to activate anti-cyst cytotoxic T cells. It would also be possible that the process of activating anti-cyst CD8^+^ cytotoxic T cells and/or initiating the anti-cyst process by the CD8^+^ cytotoxic T cells are more vulnerable to the negative environment induced by the susceptible genetic background of C57BL/6 mice to the infection than the process of activating IFN-γ-producing CD8^+^ T cells against tachyzoites and operating IFN-γ-mediated prevention of tachyzoite growth.

In relation to our findings on the capability of the HLA-A2.1 molecule to activate IFN-γ production by CD8^+^ T cells and confer a protection against cerebral tachyzoite growth, a recent study by others using synthetic peptides showed that some peptides derived from *T. gondii* molecules activate IFN-γ production of human CD8^+^ T cells obtained from HLA-A2.1^+^ individuals *in vitro* (35). Another study reported a protective effect of an immunization with a combination of peptides derived from *T. gondii* antigens against a challenge infection with the parasite in the HLA-A2.1-transgenic mice (36). However, control groups, which are CD8^+^ T cells from HLA-A2.1-negative individuals in the former study and the immunization in the WT control mice in the latter study, were not included in these studies. Therefore, in these studies it is unclear how much of the IFN-γ production by the CD8^+^ T cells and the protection against the challenge infection were eventually mediated by the antigen presentation by the HLA-A2.1.

In the present study, consistent with the potent activation of IFN-γ production by CD8^+^ T cells against *T. gondii* tachyzoites through antigen presentation by the HLA-A2.1 *in vitro*, we found that infected HLA-A2.1-transgenic mice had three time grater numbers of IFN-γ producing CD8^+^ T cells in their spleens than did infected WT mice. In addition, we identified that the greater numbers of IFN-γ-producing CD8^+^ T cells in the transgenic mice is associated with increased frequencies of the usages of TCR Vβ5.1, 5.2, Vβ8.1, 8.2, and Vβ11 in the IFN-γ-producing CD8^+^ T cells. Thus, it is likely that these TCR Vβ chains expressed on the CD8^+^ T cells contribute to recognition of *T. gondii* antigens presented by the HLA-A2.1 to activate their IFN-γ production.

In agreement with the highly enhanced capability of CD8^+^ T cells to produce IFN-γ in response to tachyzoite antigens presented by the HLA-A2.1 *in vitro* and the increased numbers of IFN-γ^+^CD8^+^ T cells in the spleens of the infected HLA-A2.1-transgenic mice, the present study identified that the efficiency of IFN-γ production in response to tachyzoites (the ratios of IFN-γ levels against tachyzoite-specific SAG1 mRNA levels) in the brain of infected HLA-A2-transgenic mice were twice higher than that of infected WT mice. The efficiencies of the expression of three effector molecules (Gbp1, NOS2, and Irgm3) of the IFN-γ-mediated protective immunity against tachyzoites were also greater in the brains of HLA-A2.1-transgenic than WT mice during reactivation of cerebral *T. gondii* infection. Therefore, CD8^+^ T cells that infiltrated into the brains of infected HLA-A2.1-transgenic mice most likely produce IFN-γ efficiently by recognizing tachyzoite antigens presented by the HLA-A2.1 and activate the IFN-γ-mediated effector mechanisms to prevent cerebral tachyzoite growth.

A direct evidence on the capability of the CD8^+^ T cells to prevent cerebral tachyzoite growth through antigen presentation by the HLA-A2.1 was provided by adoptive transfer of CD8^+^ immune T cells from infected HLA-A2.1-transgenic and WT mice to infected, T cell-deficient NSG mice expressing the HLA-A2.1. This study identified that the reactivation index (the ratios of tachyzoite-specific SAG1 mRNA levels versus bradyzoite-specific BAG1 mRNA levels) in the brains of the recipients of CD8^+^ immune T cells from the HLA-A2.1-transgenic mice were more than three times lower than those the control groups with a transfer of no T cells or WT CD8^+^ T cells. The present study also showed that the cerebral expressions of IFN-γ and the effector molecules (Gbp1, NOS2, and IDO1) against tachyzoites in the recipients of the CD8^+^ T cells from these transgenic mice were significantly increased when compared to the recipients of the WT CD8^+^ T cells. These results together clearly indicate that CD8^+^ T cells activated against *T. gondii* by antigen presentation by the HLA-A2.1 is able to confer a potent protection against reactivation of cerebral *T. gondii* infection, and the protective activity is most likely mediated by their increased production of IFN-γ and an enhancement of IFN-γ-mediated protective pathways against tachyzoites. To our knowledge, the capability of CD8^+^ T cells activated through the human HLA-A2.1 to confer a protection against cerebral tachyzoite proliferation and reactivation of *T. gondii* infection has not been reported before.

The present study identified the capability of human HLA-A2.1 to mediate a potent activation of IFN-γ production by CD8^+^ T cells against *T. gondii* tachyzoites and powerfully activate their protective activity to inhibit reactivation of cerebral *T. gondii* infection. We previously demonstrated that an adoptive transfer of CD8^+^ immune T cells from infected BALB/c mice, which are genetically resistant to cerebral infection with *T. gondii*, to infected immunodeficient mice lacking T cells is able to prevent reactivation of cerebral *T. gondii* infection in the absence of CD4^+^ T cells (15). Therefore, if we develop a vaccine that potently activates those protective CD8^+^ T cells capable of preventing reactivation of cerebral *T. gondii* infection through antigen presentation by the HLA-A2.1, it would be possible that an immunization of HIV-infected individuals expressing the HLA-A2.1 with such vaccine prior to their decreases of CD4^+^ T cell counts will be able to prevent, or at least reduce, reactivation of cerebral *T. gondii* infection in those individuals even when their CD4^+^ T cell counts become low at later time points. TE is the most common opportunistic infectious disease in the brain in AIDS patients (1, 9–11), and the HLA-A2.1 is one of the three most common MHC class I molecules in humans as mentioned earlier. Therefore, it would be important to pursue this vaccine approach to prevent or reduce the development of TE in HIV-infected individuals.

## Data availability statement

The original contributions presented in the study are included in the article/Supplementary Material. Further inquiries can be directed to the corresponding author.

## Ethics statement

The animal study was reviewed and approved by The Institutional Animal Care and use Committee.

## Author contributions

Designing research studies: YS and RM. Funding acquisition: YS. Conducting experiments and acquiring the data: RM, MA, AM, and YS. Analyzing the data: RM and YS. Writing and reviewing the manuscript: RM, MA, AM, and YS. All authors contributed to the article and approved the submitted version.

## Funding

The study was supported in part by the funding from the National Institutes of Health (AI152597, AI095032, and AI136821).

## Acknowledgments

The authors appreciate the service and assistance in flow cytometry and histological studies provided by Markey Shared Resource Facility at University of Kentucky supported by P30CA177558.

## Conflict of interest

The authors declare that the research was conducted in the absence of any commercial or financial relationships that could be construed as a potential conflict of interest.

## Publisher’s note

All claims expressed in this article are solely those of the authors and do not necessarily represent those of their affiliated organizations, or those of the publisher, the editors and the reviewers. Any product that may be evaluated in this article, or claim that may be made by its manufacturer, is not guaranteed or endorsed by the publisher.
